# Exploring the use, usefulness and ease of use of digital occupational health services: A descriptive correlational study of customer experiences

**DOI:** 10.1177/20552076241242668

**Published:** 2024-04-09

**Authors:** Sari Nissinen, Sanna Pesonen, Pauliina Toivio, Erja Sormunen

**Affiliations:** 1 3860 Finnish Institute of Occupational Health, Helsinki, Finland

**Keywords:** health services, occupational health, digital health, telemedicine, customer, patient, usefulness, ease of use

## Abstract

**Objective:**

This study examined the customer experiences of use, perceived usefulness and ease of use of digital occupational health (OH) services.

**Methods:**

A cross-sectional study based on an electronic survey was conducted between December 2022 and January 2023. A total of 9871 OH customers responded to the survey. The sample was restricted to respondents who used digital OH services (n = 7275). An analysis of variance was run to test the relationships between respondents’ characteristics and the rate of usefulness, and ease of use variables.

**Results:**

The most commonly used digital services were appointment booking, access to health information recorded by professionals and prescription renewal, and the digital services provided by physicians and nurses. Respondents expressed quite high satisfaction with the digital services, but not as much with their usefulness and ease of use. Females, individuals under 50 years of age, those with higher education, working in white-collar or managerial positions and possessing proficient information and communication technology (ICT) skills gave the most positive evaluations regarding usefulness and ease of use.

**Conclusions:**

There was a certain level of mixed experiences among respondents regarding the usefulness and ease of use of digital OH services. We can also conclude that individuals who possess the necessary ICT skills can more easily take full advantage of the available digital services. When customers are proficient in using digital services, they can confidently interact with professionals. Regardless of the user's age, gender, education or profession, it is crucial for service providers always to strive to improve the usability of digital services.

## Introduction

In Finland, the health system is organised by the public sector, which is responsible for primary and specialised care. Alongside this, there is occupational health (OH) care, which primarily offers preventive services and OH-focused medical care for the working population. The private sector plays a significant role in OH services, covering around 88% of OH providers. Additionally, employers have a role in the organisation of OH services, as they are obligated to arrange preventive OH services for their employees. Hence, OH services play a crucial role in providing healthcare services for the working population, as approximately 90% of wage earners are covered by these services. Being the healthcare provider for this demographic, OH services have a profound influence on the health and work ability of the working-age population.^1,2^

In OH services, customer work is conducted by a core multiprofessional OH team consisting of an OH physician, OH nurse, OH physiotherapist and OH psychologist. This team provides comprehensive healthcare services for customers with a focus on promoting work health and safety. In addition to preventive services, OH services include medical care services aimed at preventing work-related illnesses and disabilities. These services encompass various health check-ups, guidance on healthy working and living habits, as well as collaboration negotiations such as work ability negotiations with employees and their supervisors. Alongside the OH multidisciplinary team, public health nurses (RN) can also provide medical care services for OH customers.^3–6^

In the last decade the use of digital health services has clearly increased. For example, the necessity for medical care can be assessed through remote means such as telephone consultations or other telehealth services.^3,7,8^ Also, year 2020 brought about changes, particularly in the number of digital healthcare appointments, due to the COVID-19 pandemic.^9^ The limitations imposed on face-to-face reception services prompted healthcare professionals to swiftly adapt and cater to the needs of those working remotely or in quarantine and offer services to those who were unable to physically attend appointments to curb the spread of the virus.^10–13^

Most commonly, digital health services mean providing a service or treatment to a patient via video or telephone.^7,14^ The telephone was once the only tool of communication, but now there are also other digital transaction services, such as chat or e-mail.^8,15^ These digital health services provide an opportunity to improve the quality of care and the efficiency of service processes, while also enhancing patient satisfaction. This means that digital services need to be easy to use, patients should have easier access to get services and the exchange of information between patients and professionals should be smooth.^13,16,17^ However, in the development of digital health services, more information about the needs of customers is necessary, as the development work may often focus only on the needs of the service providers.^18^

OH service providers had been developing digital health services even before the COVID-19 pandemic,^1,9^ such as electronic appointment booking, video visits or the possibility to monitor one's own health and work ability data.^7^ With the development of digital services, OH professionals have been able to respond to the needs of their customers faster, with increased quality and a more customer-oriented approach than before.^19^ In Finland in 2021, approximately 2 million employees (90% of wage earners) were covered by OH services. During the same year, OH professionals conducted 1.4 million health examinations, 13% of which were carried out digitally, along with 1.4 million guidance visits (16% digitally), and 3.1 million medical care visits (18% digitally) for these employees.^1^ Comparing this to the Finnish public social and health care system, in 2020 the prevalence of digital health services varied among citizens’ regions, ranging from 12% to 35%.

Typically, OH customers have expressed satisfaction with the digital services provided and have had positive experiences of them. For example, video and chat consultations with physicians and psychologists have been well received by OH customers. Additionally, the option to book appointments electronically has been viewed positively. However, some may have had challenges with electronic appointment booking when a suitable time or desired professional has not been found.^9,15^

Previous studies have demonstrated a positive relationship between the perceived usefulness of digital health services and increased use.^20,21^ Furthermore, a positive attitude and motivation towards technology use has also been recognised as a key factor in the adoption and utilisation of digital health services.^22^ In addition to attitudes, having sufficient skills to use digital services has been identified as a key determinant of use, particularly among individuals beyond middle age.^22,23^ Consequently, individuals who possess good information and communication technology (ICT) skills and for whom it is easy and convenient to use digital health services are likely to find it easier to use digital health services and access their potential benefits.^24,25^ In general, OH customers have the skills necessary to use digital health services.^3^

The use of digital health services by patients, citizens and professionals has been studied.^8,14,26^ However, customers’ experiences of using digital OH services have been studied less.^15^ The aim of this study was to investigate OH customers’ perceptions of the use, usefulness and ease of use of digital OH services and to identify factors associated to the usefulness and ease of use of digital OH services. The study results offer a better understanding of customers’ experiences with digital OH services. The results benefit OH providers in developing and improving the usability and quality of digital services.

The following research questions were asked:

RQ1: How do customers use digital occupational health services?
RQ2: How satisfied the respondents are to occupational health services and to digital occupational health services generally?
RQ3: How do customers perceive the usefulness and ease of use of digital occupational health services?
RQ4: Are there differences in perceived usefulness and ease of use among background variables?
RQ5: Which factors are associated with the perceived usefulness and ease of use?

## Materials and methods

### Research design

A cross-sectional and a descriptive correlation design following Campbell and Stanley^27^ was used.

### Participants and data collection

The study was conducted through an online survey in Finland between December 2022 and January 2023. Five organisations (representing employee and entrepreneurs associations, as well as workplaces) participated in the study. The study population included all members and employees of the organisations involved in this study. A contact person from each participating organisation sent a link to the survey via email to all their members and employees. One reminder was sent to encourage responses.

Written consent to the study was not requested from participants. In Finland, answering the survey is primarily considered an indication of consent to participate in the research and to allow the use of the material in the research. All participants were provided with a formal covering letter explaining the background of the study, that a participation in the study is completely voluntary and anonymous, and that a responding to the questionnaire is interpreted as informed consent to take part in the study. Consent to use the responses for scientific research was requested in the survey. We confirm that the Ethical Board of the Finnish Institute of Occupational Health had approved this content process.

**Figure 1.**
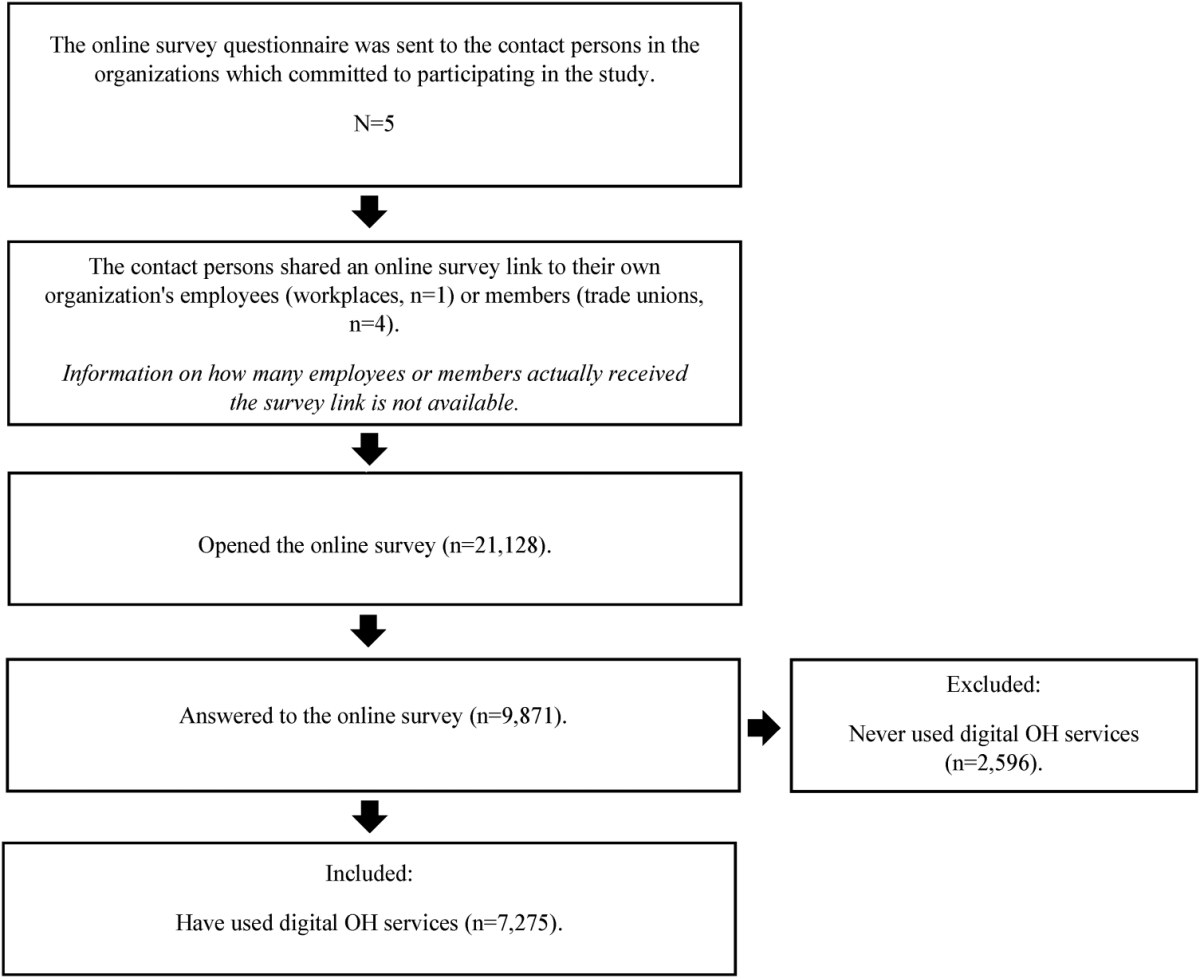
The flowchart of participants.

In total, 9871 OH customers participated in the study. Among them, 2596 were excluded as they indicated they had never used digital OH services. Consequently, the final count of participants was 7275. Table 1 presents the distribution of respondents’ gender, age, level of educational degree, perceived ICT skills, area of residence, occupation, a workplace size, and OH provider. It is not possible to determine the exact response rate for the study since accurate information regarding the number of individuals who received the survey is unavailable. Out of the 21,128 respondents who opened the survey link, 46.7% completed the survey. Figure 1 illustrates the participants’ flowchart.

**Table 1.** Respondent's characteristics.

Characteristics	Have use digital occupational health services		Total
	Yes, n (%)	No, n (%)	n (%)
Gender			
Female	5704 (79)	1991 (78)	7695 (79)
Male	1501 (21)	562 (22)	2063 (21)
Total	7205 (100)	2553 (100)	9758 (100)
Age			
≤50 years	2503 (35)	740 (18)	3243 (32)
51–60 years	3503 (48)	1277 (32)	4780 (49)
Over 61 years	1264 (17)	2017 (50)	1833 (19)
Total	7270 (100)	4034 (100)	9856 (100)
Level of educational degree			
Lower level (public, primary, high, or vocational school)	3777 (52)	1541 (60)	5318 (54)
Upper level (vocational college, vocational university, or university)	3478 (48)	1037 (40)	4515 (46)
Total	7255 (100)	2578 (100)	9833 (100)
Perceived ICT skills			
Good	3741 (52)	1038 (40)	4779 (49)
Moderate or weak	3502 (48)	1534 (60)	5036 (51)
Total	7243 (100)	2572 (100)	9815 (100)
Area of residence			
Southern Finland	4187 (58)	1364 (53)	5551 (57)
Eastern Finland	658 (9)	248 (10)	906 (9)
Western Finland	1409 (20)	655 (26)	2064 (21)
Northern Finland	963 (13)	296 (12)	1259 (13)
Total	7217 (100)	2563 (100)	9780 (100)
Profession			
Management (director or supervisor)	459 (6)	151 (6)	610 (6)
Occupational safety representative or manager	287 (4)	60 (2)	347 (4)
White-collar worker	795 (11)	193 (8)	988 (10)
Blue-collar worker	5645 (79)	2149 (84)	7794 (80)
Total	7186 (100)	2553 (100)	9739 (100)
Workplace size			
Entrepreneur or under 10 employees	1136 (16)	554 (22)	1690 (17)
10–49 employees	2696 (37)	976 (38)	3672 (38)
50–249 employees	1447 (20)	446 (17)	1893 (19)
250 or more employees	1938 (27)	586 (23)	2524 (26)
Total	7217 (100)	2559 (100)	9776 (100)
Occupational health provider			
Private sector	4821 (67)	1337 (52)	6158 (63)
Public sector or employer's occupational health centre	1464 (20)	718 (28)	2182 (22)
Something else/can't say/no occupational health care available	966 (13)	519 (20)	1485 (15)
Total	7251 (100)	2574 (100)	9825 (100)

ICT: information and communication technology.

### Survey development

The survey included questions on respondents’ use of digital OH services (with predefined answer options): how often they have used digital OH services, what digital OH services they have used and which professionals’ digital OH services they have used. In addition, there were questions on their satisfaction with both OH services and digital OH services (on a scale of 4–10). Also, the survey included questions on the usefulness and ease of use of digital OH services, which were measured using different statements. These statements based on the Davis's Technology Acceptance Model.^28^ Respondents were asked to select how they felt about the following statements concerning the usefulness of digital OH services: They (a) improve the quality of the service; (b) give me greater control over my illness; (c) give me greater control over my work ability; (d) help me get the help I need from OH professionals even faster; and (e) help me get the information I need about health, illness or work ability even faster. Respondents were asked to select how they felt about the following statements concerning the ease of use of digital OH services: (a) learning to use digital OH services is easy for me, (b) it is easy to use digital OH services, (c) I can use digital OH services flexibly according to my needs, (d) the use of digital OH services is clear and (e) I can easily find instructions for using digital OH services. A 5-point Likert scale was used to respond to the statements (1 = completely disagree to 5 = completely agree). Before the data collection phase, the readability and comprehensibility of the survey content was checked by five specialists (with a background in OH customer services). Their comments were used to prepare the final version of the survey. Appendix 1 presents the content of the originally Finnish-language questionnaire translated into English. The authors have permission to publish the translated questionnaire.

### Data analysis

The survey data was analysed descriptively using frequency analysis (RQ1-RQ3) in SPSS Statistics 27 software. The key survey variables, which investigate the usefulness and ease of use of digital OH services, were aggregated into sum variables, and their associations with respondents’ gender, age, education, ICT skills, area of residence, occupation, workplace size and OH provider skills were examined. Prior to forming the sum variables, the consistency of the variables was assessed using Cronbach's alpha coefficient, resulting in values of 0.921 for the usefulness sum variable and 0.755 for the ease of use sum variable.

Group-level differences in the sum variables based on the TAM model^28^ were examined using one-way analysis of variance (ANOVA) (RQ4), which compares the means of several groups and determines whether there are statistically significant differences between these groups. For dichotomous variables, we used the T-test and Scheffe's post hoc test for others. A *p*-value <0.001 indicates a statistically significant result. We chose a statistical significance level of <0.001 in order to have a stricter criterion for assessing the significance of the results and the risk of false positive results lower particularly because of the large sample size. This helps to obtain more reliable and practically significant results. Additionally, we calculated the retrospective effect size for mean differences at a power level of 0.8 and a *p*-value of 0.001 for a sample comprising 7275 observations, and we obtained a value of 0.048. A larger effect size indicates a stronger or more meaningful practical significance of the observed difference of means.

Linear regression analysis was employed to examine the relationship between multiple explanatory variables and perceived usefulness and ease of use (RQ5). In the first model, the explanatory variables were background variables (Model 1), and in the second model, the satisfaction with OH services variable, along with the satisfaction with digital OH services variable, were included (Model 2). Before conducting linear regression analysis, the relationships between variables were examined using Pearson's correlation. The fulfilment of the conditions for linear regression analysis was ensured by checking the correlations between variables and their variance inflation factors (VIFs): correlations were not high, and VIF values varied between 1 and 2.

## Results

### Participants

Characteristics of the participants (N = 7275) are presented in Table 1. Participants were predominantly women (n = 5704/7205, 79%), blue-collar workers (n = 5645/7186, 79%) and aged 51–60 years (n = 3503/7270, 48%). Over half of respondents had completed a lower level educational degree (n = 3777/7255, 52%). Additionally, slightly over half of respondents rated their ICT skills as good (n = 3741/7243, 52%).

### The use of digital occupational health services

**Figure 2.**
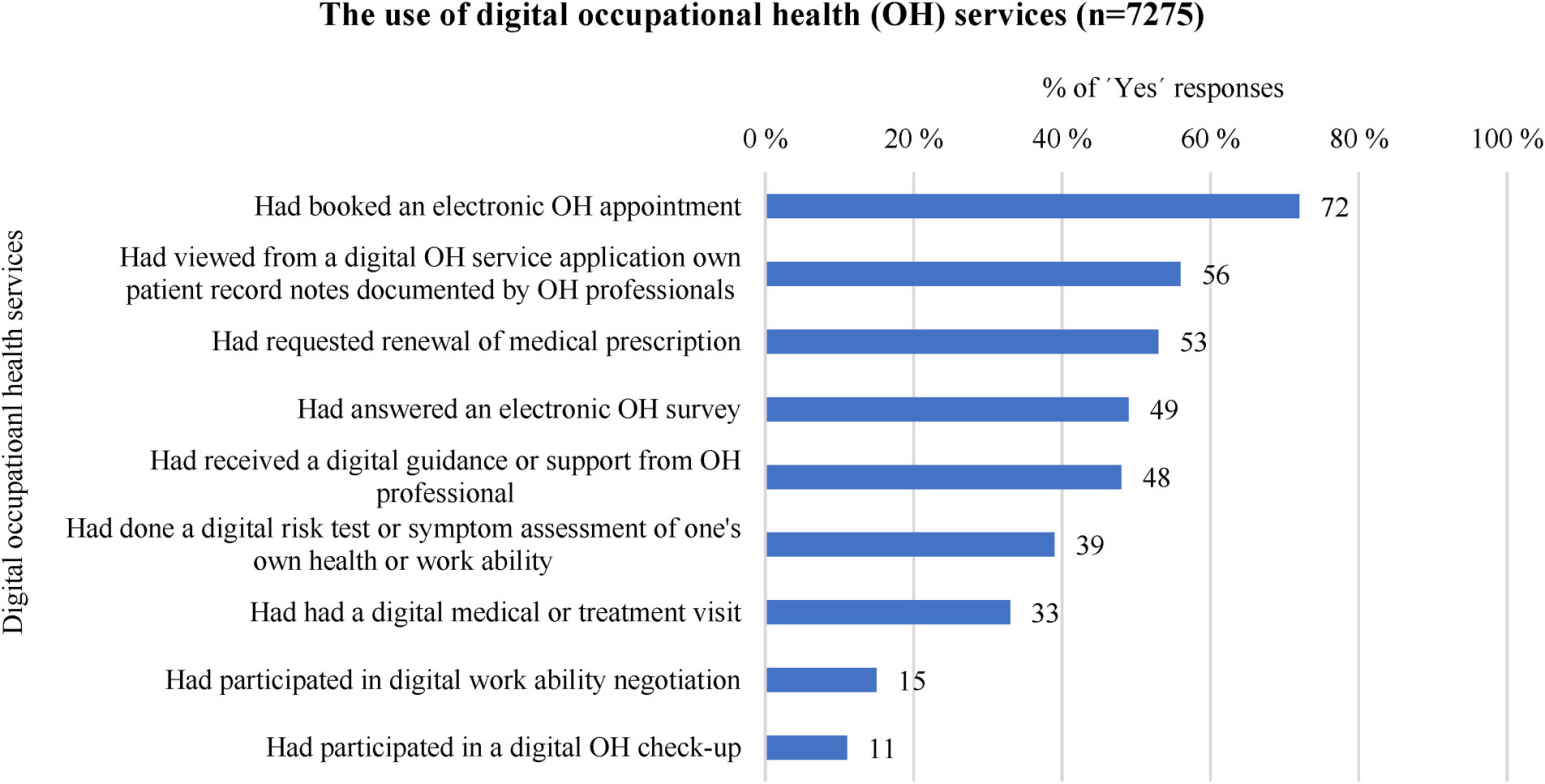
Respondents’ use of digital occupational health services (n = 7275, % of ‘Yes’ responses). OH: occupational health.

In Figure 2, the use of digital OH services in different digital services offered to customers is described. The most used service by respondents (n = 7275) was the option of electronic OH appointment booking, as approximately three-quarters (n = 5215, 72%) reported having made use of it. Over half of respondents (n = 4092, 56%) indicated that they had viewed from the digital OH service application their own medical care visit notes recorded by OH professionals. Almost a similar number of respondents (n = 3865, 53%) had requested prescription renewals through digital OH services. One-third of respondents (n = 2402, 33%) reported having used a digital medical care visit. The least frequently mentioned activities among respondents were digital work ability negotiations (n = 1079, 15%) and digital health check-ups (n = 814, 11%).

**Figure 3.**
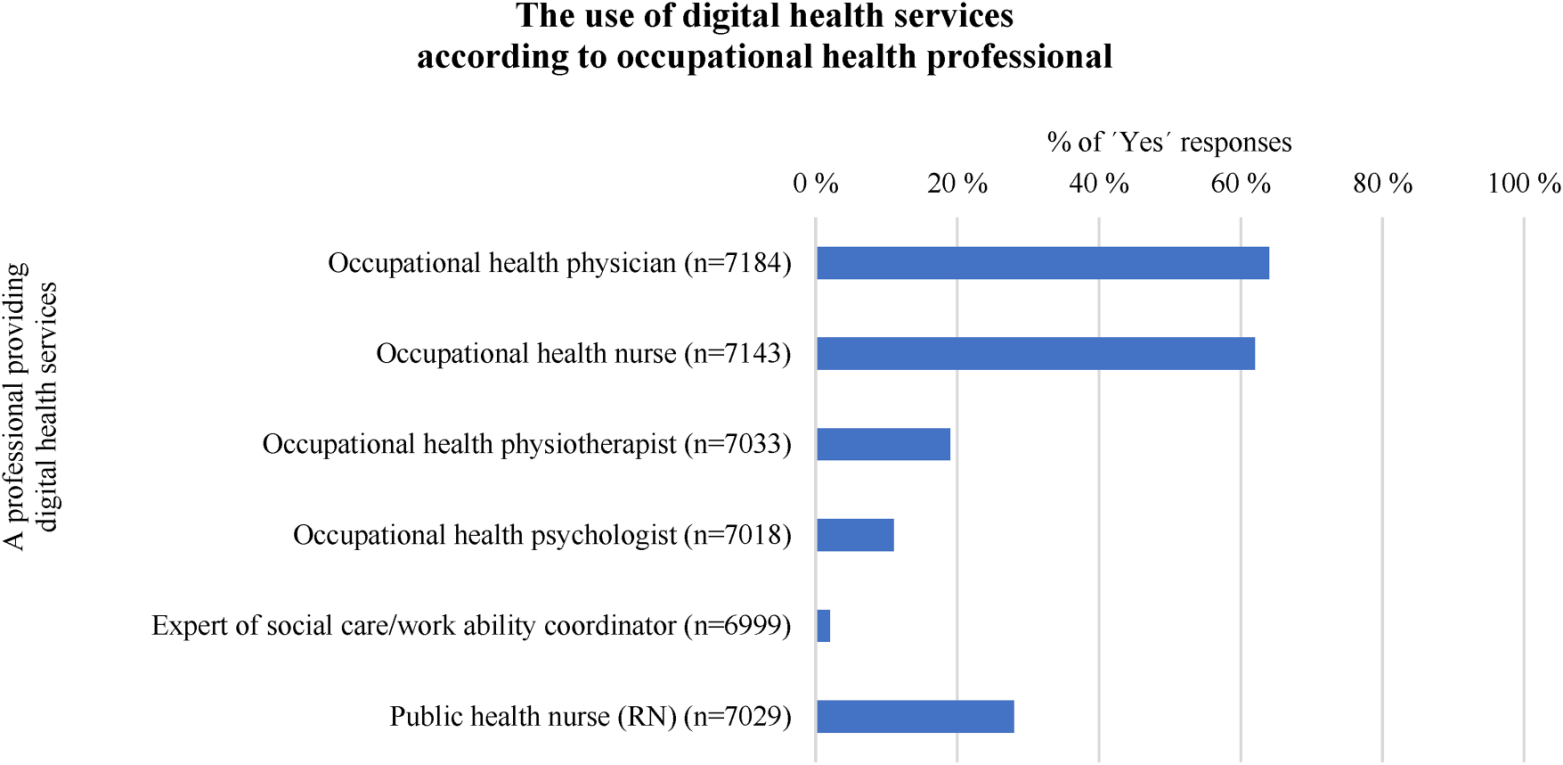
The relative proportions of respondents who have used the digital OH services of different professionals (% of ‘Yes’ responses).

Respondents were asked to indicate which OH professional's digital services they had used. Clearly, the majority had used the digital OH services provided by an OH physician (n = 4587/7184, 64%) and an OH nurse (4426/7143, 62%). Over a quarter of respondents had used the digital services of a public health nurse (RN) (1981/7029, 28% of respondents). Figure 3 presents the relative proportions of respondents who have used the digital OH services of different professionals.

Respondents (n = 7275) primarily used their phones for communication with OH services (n = 6180, 85%). Four out of 10 had used a chat service (n = 3041, 42%) and one-third had used email (n = 2422, 33%) for their interactions with OH services. Respondents (n = 7275) were also asked to rate their satisfaction with the OH services. Overall, they gave a rating of 7.2 (on a scale of 4–10) for OH services and 7.4 for digital OH services.

### Perceived usefulness and ease of use of digital occupational health services

According to the results describing the usefulness and ease of use of digital OH services, respondents often chose the neutral option of ‘Neither agree nor disagree’ (25–40% of respondents). On the other hand, for many statements, there were nearly equal numbers of respondents who disagreed and agreed. The results for respondents’ attitude statements regarding perceived usefulness and ease of use of digital OH services are presented in Table 2.

**Table 2.** Respondents’ attitude statements regarding perceived usefulness and ease of use of digital occupational health services (% of respondents).

	Completely disagree, n (%)	Somewhat disagree, n (%)	Neither agree nor disagree, n (%)	Somewhat agree, n (%)	Completely agree, n (%)
The usefulness of digital occupational health (OH) services					
Using digital OH services improve the quality of the service (n = 7178).	923 (13)	1350 (19)	2475 (34)	1835 (26)	595 (8)
Using digital OH services give me greater control over my illness (n = 7168).	1105 (15)	1210 (17)	2891 (40)	1495 (21)	467 (7)
Using digital OH services give me greater control over my work ability (n = 7166).	1125 (16)	1265 (18)	2837 (39)	1487 (21)	452 (6)
Using digital OH services help me get the help I need from OH professionals even faster (n = 7162).	830 (12)	915 (13)	1800 (25)	2447 (34)	1170 (16)
Using digital OH services help me get the information I need about health, illness, or work ability even faster (n = 7163).	916 (13)	1013 (14)	2548 (36)	1963 (27)	723 (10)
The ease of use of digital occupational health (OH) services					
Learning to use digital OH services is easy for me (n = 7183).	402 (6)	777 (11)	1852 (25)	2700 (38)	1452 (20)
It is easy to use digital OH services (n = 7176).	454 (6)	860 (12)	1851 (26)	2598 (36)	1413 (20)
I can use digital OH services flexibly according to my needs (n = 7174).	625 (9)	868 (12)	2160 (30)	2244 (31)	1277 (18)
The use of digital OH services is clear (n = 7163).	558 (8)	1057 (15)	2289 (32)	2165 (30)	1094 (15)
I can easily find instructions for using digital OH services (n = 7165).	719 (10)	1230 (17)	2586 (36)	1814 (25)	816 (12)

OH: occupational health.

When comparing those who disagreed with those who agreed, the difference in favour of those who disagreed was in the statement ‘Using digital occupational health services gives me greater control over my work ability’ (Completely disagree 16%, Somewhat disagree 18%, Somewhat agree 21%, Completely agree 6%). Conversely, comparing those who agreed with those who disagreed, the difference in favour of those who agreed was in the statement ‘Using digital occupational services helps me get the help I need from OH professionals even faster’ (Completely agree 16%, Somewhat agree 34%, Somewhat disagree 13%, Completely disagree 12%).

In the results describing the ease of use of digital OH services, there were more respondents who agreed than disagreed for all statements. The clearest differences were observed in the statements ‘Learning to use digital occupational services is easy for me’ (Completely agree 20%, Somewhat agree 38%, Somewhat disagree 11%, Completely disagree 6%) and ‘It is easy to use digital occupational health services’ (Completely agree 20%, Somewhat agree 36%, Somewhat disagree 12%, Completely disagree 6%).

### Differences in perceived usefulness and ease of use of digital occupational services

We performed an ANOVA to assess the significance of individual background variables (gender, age, level of education, perceived ICT skills, area of residence, occupation, workplace size and OH provider) in explaining perceived usefulness and perceived ease of use regarding digital OH services. The results of this analysis are presented in Table 3.

**Table 3.** ANOVA results of the differences between perceived usefulness and ease of use and respondents’ characteristic groups.

	Perceived usefulness				Perceived ease of use			
	n	Mean (SD)	F (df)	*P*	n	Mean (SD)	F (df)	*P*
Gender	7105		F(1, 7103) = 18.636	.000	7114		F(1, 7112) = 27.869	.000
Female	5630	3.04 (1.01)*			5636	3.40 (1.01)*		
Male	1475	2.91 (1.03)*			1478	3.25 (1.02)*		
Age	7170		F(2, 7167) = 34.796	.000	7178		F(2, 7175) = 88.345	.000
Under 50 years	2471	3.15 (0.99)^a^			2475	3.58 (1.00)^a^		
51–60 years	3458	2.96 (1.02)^b^			3462	3.30 (1.01)^b^		
Over 61 years	1241	2.89 (1.02)^b^			1241	3.15 (1.02)^c^		
Level of educational degree	7154		F(1, 7152) = 25.842	.000	7162		F(1, 7160) = 40.991	.000
Lower level (public, primary, high, or vocational school)	3728	2.95 (1.01)*			3728	3.30 (1.02)*		
Upper level (vocational college, vocational university, or university)	3426	3.08 (1.02)*			3434	3.50 (1.01)*		
Perceived ICT skills	7144		F(1, 7142) = 72.409	.000	7152		F(1, 7150) = 325.222	.000
Good	3691	3.11 (1.03)*			3698	3.58 (1.01)*		
Moderate or weak	3453	2.91 (0.99)*			3454	3.15 (0.98)*		
Area of residence	7123		F(3, 7119) = 4.692	.003	7131		F(3, 7127) = 5.245	.001
Southern Finland	4141	3.04 (1.01)			4144	3.40 (1.02)		
Eastern Finland	649	3.02 (1.02)			650	3.36 (1.01)		
Western Finland	1385	2.93 (1.03)			1388	3.28 (1.03)		
Northern Finland	948	3.01 (1.00)			949	3.38 (0.98)		
Occupation	7086		F(3, 7082) = 22.062	.000	7094		F(3, 7090) = 34.769	.000
Management position (director or supervisor)	449	3.31 (0.98)^a^			452	3.73 (1.01)^a^		
Occupational safety representative or manager	283	3.06 (1.03)			283	3.49 (1.02)		
White-collar worker	784	3.16 (0.99)^a^			786	3.55 (0.96)^a^		
Blue-collar worker	5570	2.97 (1.01)^b^			5573	3.31 (1.02)^b^		
Workplace size	7122		F(3, 7118) = 4.079	.007	7130		F(3, 7126) = 7.194	.000
Entrepreneur or under 10 employees	1122	3.05 (1.03)			1122	3.31 (1.01)		
10–49 employees	2659	2.96 (1.02)			2661	3.32 (1.02)		
50–249 employees	1427	3.06 (0.98)			1427	3.45 (1.02)		
250 or more employees	1914	3.04 (1.03)			1920	3.42 (1.01)		
Occupational health provider	7154		F(2, 7151) = 39.202	.000	7163		F(2, 7160) = 81.352	.000
Private sector	4767	3.09 (1.00)^a^			4774	3.48 (1.01)^a^		
Public sector or employers’ own occupational health centre	1446	2.86 (1.03)^b^			1442	3.16 (1.00)^b^		
Something else	941	2.87 (1.03)^b^			947	3.16 (1.00)^b^		

Note: Data are mean ± standard deviation, *p*-value from one-way ANOVA.

The significant pair difference based on T-test: *(*p*-value <0.001 is considered to be significant).

The significant intergroup difference based on Scheffe's multiple comparison test: a > b > c (*p*-value <0.001 is considered to be significant).

ICT: information and communication technology.

Regarding the perceived usefulness of digital OH services, the ANOVA test revealed statistically significant differences (*p*-value <0.001) among gender (F(1, 7103) = 18.636, *p* = 0.000), age (F(2, 7167) = 34.796, *p* = 0.000), level of educational degree (F(1, 7152) = 25.842, *p* = 0.000), perceived ICT skills (F(1, 7142) = 72.409, *p* = 0.000), occupation (F(3, 7082) = 22.062, *p* = 0.000) and OH provider (F(2, 7151) = 39.202, *p* = 0.000). In terms of the ease of use of digital OH services, the ANOVA test indicated highly significant statistical differences (*p*-value <0.001) among all groups, except for the respondents’ area of residence (*p* = 0.001). To further explore the associations between respondents’ characteristics and their perception of the usefulness and ease of use of digital OH services, post hoc tests (T-test) were conducted (see Table 3).

The results revealed that in evaluating the usefulness of digital OH services, there were significant differences between female ratings (mean 3.04) and male ratings (mean 2.91), as well as between the ratings of respondents with good ICT skills (mean 3.11) and those with moderate or weak ICT skills (mean 2.91). Additionally, the ratings of respondents with upper level educational degrees (mean 3.08) significantly differed from those with lower level educational degrees (mean 2.95).

Similar group differences were observed for the perceived ease of use of digital OH services (see Table 3). There were significant differences between female ratings (mean 3.40) and male ratings (mean 3.25), as well as between the ratings of respondents with good ICT skills (mean 3.58) and those with moderate or weak ICT skills (mean 3.15). Additionally, ratings from respondents with upper level educational degrees (mean 3.50) significantly differed from those with lower level educational degrees (mean 3.30).

The post hoc tests (Scheffe) revealed significant differences (*p* < 0.001) in the evaluation of the usefulness of digital OH services among different groups (as shown in Table 3). Especially respondents under 50 years old (mean 3.15) rated the usefulness significantly higher compared to those aged 51–60 years old (mean 2.96) or over 61 years old (mean 2.89). Additionally, blue-collar workers (mean 2.97) reported significantly lower usefulness ratings compared to white-collar workers (mean 3.16) and individuals in management positions (mean 3.31). Similar group differences were observed for the perceived ease of use of digital OH services. In other words, respondents under 50 years old (mean 3.58) rated the ease of use significantly higher compared to those aged 51–60 years old (mean 3.30) or over 61 years old (mean 3.15). Moreover, there were significant differences between respondents aged 51–60 years old and over 61 years old. Furthermore, respondents whose OH provider was in the private sector rated the usefulness of OH services (mean 3.09) and ease of use (mean 3.48) significantly higher than those whose OH provider was in the public sector or their employers’ own OH centre (mean between 2.86–2.87) or was something else (mean 3.16). There were no significant differences between respondents’ area of residence or workplace size.

In the retrospective estimation of the effect size, the calculated effect size for mean differences was 0.048. Based on the results from Table 3, it was observed that the sample sizes were sufficient for all other variables except for residential area, workplace size and healthcare provider.

### Factors associated with perceived usefulness and perceived ease of digital occupational health services

To test associations between respondents’ characteristics and perceived usefulness and ease of use, we used linear regression analysis. The variables gender, age, level of educational degree, perceived ICT skills, area of residence, profession, workplace size and OH provider were included in Model 1 for this regression analysis. In Model 2, we added the variables satisfaction with OH services and satisfaction with digital OH services. The linear regression results for the usefulness of digital OH services are presented in Table 4, and the results for the ease of use of digital OH services are presented in Table 5.

**Table 4.** Multivariable linear regression analyses for factors associated to the perceived usefulness of digital occupational health services.

	Usefulness of digital OH services									
	Model 1 characteristics					Model 2 (+satisfaction with OH services)				
	B (95% CI)	SE	Β	t	*p*	B (95% CI)	SE	β	t	*p*
Gender	0.155 (0.094, 0.215)	0.031	0.061	5.032	<0.001	0.135 (0.079, 0.190)	0.028	0.053	4.781	<0.001
Age	−0.106 (−0.141, −0.071)	0.018	−0.073	−5.953	<0.001	−0.117 (−0.149, −0.085)	0.016	−0.080	−7.149	<0.001
Level of educational degree	0.042 (−0.008, 0.092)	0.026	0.021	1.643	0.100	0.022 (−0.024, 0.068)	0.023	0.011	.926	0.355
Perceived ICT skills	−0.124 (−0.175, −0.073)	0.026	−0.061	−4.773	<0.001	−0.134 (−0.180, −0.087)	0.024	−0.066	−5.601	<0.001
Area of residence	−0.035 (−0.056, −0.014)	0.011	−00.39	−3.253	0.001	−0.021 (−0.040, 0.002)	0.010	−0.023	−2.127	0.033
Profession	−0.083 (−0.113, −0.053)	0.015	−0.068	−5.403	<0.001	−0.046 (−0.073, −0.018)	0.014	−0.038	−3.267	0.001
Workplace size	−0.002 (−0.025, 0.021)	0.012	−0.002	−.150	0.880	0.006 (−0.015, 0.027)	0.011	0.006	.554	0.579
OH provider	−0.121 (−0.155, −0.088)	0.017	−0.085	−7.083	<0.001	−0.059 (−0.090, −0.028)	0.016	−0.041	−3.739	<0.001
Satisfaction with occupational health services						0.196 (0.181, 0.211)	0.008	0.344	25.029	<0.001
Satisfaction with digital occupational health services						0.046 (0.032, 0.060)	0.007	0.089	6.513	0.000
*R*	0.033					0.193				
*R* ^2^	0.032					0.191				
*F*	29.390					163.793				

ICT: information and communication technology; OH: occupational health.

**Table 5.** Multivariable linear regression analyses for factors associated to the perceived ease of use of digital occupational health services.

	Ease of use of digital OH services									
	Model 1 characteristics					Model 2 (+satisfaction with OH services)				
	B (95% CI)	SE	β	t	*p*	B (95% CI)	SE	β	t	*p*
Gender	0.192 (0.134, 0.251)	0.030	0.076	6.421	>0.001	0.175 (0.12, 0.23)	0.028	0.069	6.255	>0.001
Age	−0.154 (−0.188, −0.120)	0.017	−0.106	−8.844	>0.001	−0.163 (−0.195, −0.131)	0.016	−0.111	−10.003	>0.001
Level of educational degree	0.016 (−0.033, 0.065)	0.025	0.008	0.655	0.513	−0.004 (−0.050, 0.042)	0.023	−0.002	−0.174	0.862
Perceived ICT skills	−0.322 (−0.371, −0.272)	0.025	−0.158	−12.647	>0.001	−0.330 (−0.376, −0.284)	0.024	−0.162	−13.927	>0.001
Area of residence	−0.026, (−0.047, −0.006)	0.010	−0.029	−2.511	0.012	−0.015 (−0.034, 0.004)	0.010	−0.016	−1.506	0.132
Profession	−0.091 (−0.120, −0.062)	0.015	−0.075	−6.120	>0.001	−0.059 (−0.086, −0.031)	0.014	−0.048	−4.211	>0.001
Workplace size	0.011 (−0.011, 0.034)	0.012	0.012	0.982	0.326	.018 (−0.003, 0.039)	0.011	0.018	1.674	0.094
OH provider	−0.169 (−0.202, −0.136)	0.017	−0.119	10.146	>0.001	−0.116 (−0.146, 0.085)	0.016	−0.081	−7.408	>0.001
Satisfaction with occupational health services						0.169 (0.153, 0.184)	0.008	0.295	21.698	>0.001
Satisfaction with digital occupational health services						0.046 (0.032, 0.184)	0.007	0.089	6.574	>0.001
*R*	0.080					0.204				
*R* ^2^	0.079					0.203				
*F*	75.151					176.567				

ICT: information and communication technology; OH: occupational health.

The results show that the explanatory power of the model increases in Model 2 than Model 1 for both perceived usefulness (Model 1 R^2^ = .0032, Model 2 R^2^ = .191) and ease of use (Model 1 R^2^ = .079, Model 2 R^2^ = .203). The results also indicate that all variables, except level of educational degree, area of residence and workplace size were statistically significant predictors of both perceived usefulness and ease of use. When satisfaction with OH services and satisfaction with digital services were included in models, only the profession disappeared in perceived usefulness of digital OH services.

## Discussion

The present study examined OH customers’ use of digital OH services. Also, this study examined OH customers’ experiences of the usefulness and ease of use of digital OH services and tried to identify potential factors that may influence the perceived usefulness and ease of use of these services.

We found that electronic appointment booking, viewing personal medical care visit notes and requesting prescription renewals through digital services were the most popular services and were used by over half of respondents. A third of respondents also reported using digital medical care visits. The results were similar to the Kyytsönen & Vehko^7^ study that investigated citizens’ use of electronic health services in Finland. Digital work ability negotiations and health check-ups were clearly less commonly used by respondents. On the other hand, even in traditional form (i.e. non-digital form) OH customers generally take a part of work ability negotiations and health check-up less frequently compared to other services.

Respondents were quite satisfied with OH services and slightly more satisfied with digital OH services. Similarly, Kyytsönen et al.^3^ have found that OH customers are content with digital OH services. For instance, booking appointments at one's convenience and participating in remote meetings from home without the need to travel are perceived as positive features. However, experiences are generally negative if suitable appointment times or familiar physicians cannot be found, or if there are technical challenges during video consultations.^15^ It has to be said, as Verma & Kerrison^29^ have shown, those who use digital health services are generally content with them. On the other hand, Verma & Kerrison^29^ have noted that digital health services are more suitable for follow-up visits that have been preceded by an earlier face-to-face appointment and should not be used for initial visits to healthcare professionals or for managing new symptoms or illnesses.

According to the results, when communicating with OH professionals, most respondents used a telephone. Customers often preferred using the telephone^30^ and using the telephone should not be entirely replaced by other digital services.^23^ Also, chat and email were commonly used for interacting with OH professionals. Usually, OH customers find using a chat useful and simple to use.^15^ On the other hand, a chat is often perceived as the fastest way to contact a professional, regardless of the urgency of the matter.^31^ Conversely, it is strongly discouraged to use email for personal matters, and healthcare professionals typically prohibit its use for that, especially if it is unsecured email.^32^

The study's findings indicated that well over half of respondents (64%) had used digital services provided by OH physicians, and a similar percentage (62%) had also used digital services offered by OH nurses. Additionally, more than a quarter of respondents had made use of digital services delivered by public health nurses (RN) working in OH services. Digital solutions can be seen as equalising the availability of services among customers. Video appointments were especially seen as enabling customer participation when distances are long or when it is difficult for the customer to travel to a location. On the other hand, digitalisation may weaken the equality of access to services. For example, all customers might not be able to afford a smartphone or computer.^30,33^

In this study, the results of OH customers’ perceived usefulness and ease of use of digital OH services varied, and there was a certain level of mixed sentiment among respondents. However, further analyses with post hoc tests provided more detailed insights into respondents’ characteristics and their perception of the usefulness and ease of use of digital OH services. Additionally, the subsequent analysis revealed that customer satisfaction with OH services also plays a crucial role in influencing the perceived usefulness and ease of use of digital OH services.

Firstly, according to the results, there were significant differences in the ratings between females and males, with females rating the usefulness and ease of use of digital OH services higher than males. This indicates that gender plays a role in how individuals perceive the usefulness and ease of use of digital OH services, with females generally finding them slightly more useful and easier to use. However, in accordance with Woolley et al. (2023)^34^ and Mold et al. (2019),^35^ the previous studies are not so conclusive on the differences between genders.

Secondly, respondents with good ICT skills rated the usefulness and ease of use significantly higher compared to those with moderate or weak ICT skills. This highlights the importance of ICT skills in influencing individuals’ perceptions of the usefulness and ease of use of digital services. Those with better ICT skills are more likely to perceive OH services as useful and easy to use. Previous studies point to the same conclusion that digital health services are often considered useful and easy to use by those individuals who have the skills necessary for using digital services,^23^ and for whom the use is the easiest and most convenient.^24^ However, users’ limited ICT skills might have hindered their use of digital health services.^16,25,36^

Also, the results showed that there were notable differences in the ratings between respondents with higher educational degrees and those with lower educational degrees. Education level seems to influence individuals’ perceptions of the usefulness and ease of use of digital OH services, with higher educational degrees correlating with more positive ratings. The findings align with a recent review wherein individuals with higher education levels are more likely to use digital health services compared to those with lower educational levels.^34^

Moreover, younger people (under 50 years old) rated the usefulness and ease of use significantly higher compared to older age groups (aged 51–60 years old and over 61 years old). These results suggest that younger people have a more positive perception of the usefulness and ease of use of digital OH services compared to older age groups. The results are in line with previous studies.^23,33,35,37^ However, it is noteworthy, as highlighted by Kainiemi et al. (2023),^24^ that as individuals age, they will inevitably face challenges in using digital services unless the development of these services explicitly considers age-related needs.

Furthermore, occupation was found to impact individuals’ perceptions. Blue-collar workers reported significantly lower ratings of usefulness and ease of use compared to white-collar workers and those in managerial positions. This suggests that occupation influences how individuals perceive the usefulness and ease of use of digital OH services.

Lastly, respondents whose OH provider was in the private sector gave higher ratings for the usefulness and ease of use of OH services compared to those whose OH provider was in the public sector, their employers’ own OH centre, or something else. It's important to note that almost all OH providers in Finland are in the private sector.^1^

All in all, according to this study, the most commonly used digital OH services were appointment booking, access to health information recorded by professionals and prescription renewal, as well as the digital services provided by OH physician, OH nurse and public health nurses (RN). Generally, respondents expressed quite high satisfaction with the digital services provided in OH services, but not as much with their usefulness and ease of use. Respondents who gave the most positive evaluations regarding the usefulness and ease of use of the digital OH services were females, individuals under 50 years of age, those with higher education, working in white-collar or managerial positions and those with proficient ICT skills.

### Strengths and limitations

This was a descriptive correlational study whose aim was to find associations between different variables but not to draw causal conclusions. The research methods employed in the study were suitable for this study, providing answers to the research questions and generating new information in line with the research objectives. However, this study has both strengths and weaknesses.

One of the strengths is the extensive sample size, which ensures a substantial number of observations available for analysis, making the data highly representative. This feature enhances confidence in the accuracy and generalisability of the results. Another strength lies in its timeliness, considering the rapid growth of digital health services. Additionally, calculating the effect size was beneficial in the study because it provided an objective measure of the magnitude of observed differences. The effect size allowed for comparisons between different variables and aided in identifying clinically significant associations. Furthermore, we have provided a cross-sectional snapshot of customers’ adoption of digital OH services and the factors associated with their perceived usefulness and ease of use.

The first limitation of this study is the predominant participation of one gender, potentially impacting the generalisability of the findings. A more balanced representation of both men and women is necessary. Another limitation is that the study is based on self-assessments for data collection. Nevertheless, certain valuable information can only be obtained through surveys, as was the case in this study. The third limitation lies in the unavailability of data on non-respondents. Additionally, despite obtaining statistically highly significant results in the study, due to the low explanatory power, caution should be exercised in interpreting the findings, and the models cannot be used to make highly reliable prediction.

The practicality of the Davis's TAM model^28^ for research has been criticised. However, it has been acknowledged that despite its age and the fact that it was not originally formulated to fit healthcare technologies, the model is suitable for studying the acceptance of digital healthcare.^38^ Therefore, the study benefited from the widely recognised TAM model^28^ that was well suited to this study as well as facilitating the comparability of the research findings with similar studies. However, further research on the customer experience of digital OH services is needed.

## Conclusions

This study provided new insights into customers’ experiences of the use, perceived usefulness and ease of use of digital OH services. The most commonly used digital services in OH services were electronic appointment booking, viewing one's own health information and renewing a prescription. Additionally, digital services provided by OH physicians and OH nurses were used the most. Also, on the basis of the study results, it can be concluded that there was a certain level of mixed experiences among respondents regarding the usefulness of digital OH services. While opinions vary, there was quite a general consensus that these services can facilitate quicker access to OH professionals. The positive perception of faster accessibility also highlighted the potential benefits of digital OH services in improving efficiency. Additionally, respondents generally found digital OH services easy to use, although there were also some who disagreed. We can also conclude that individuals who possess the necessary ICT skills can more easily take full advantage of the available digital OH services. When customers are proficient in using digital OH services, they can confidently interact with OH professionals, potentially leading to increased overall satisfaction with all OH services. Also, regardless of the user's age, gender, education or profession, it is crucial for OH providers always to strive to improve the usability of digital services. Future research could delve deeper into aspects of customer satisfaction and explore factors contributing to the perceived usefulness and ease of use of digital OH services.

## Supplemental Material

sj-docx-1-dhj-10.1177_20552076241242668 - Supplemental material for Exploring the use, usefulness and ease of use of digital occupational health services: A descriptive correlational study of customer experiences Supplemental material, sj-docx-1-dhj-10.1177_20552076241242668 for Exploring the use, usefulness and ease of use of digital occupational health services: A descriptive correlational study of customer experiences by Sari Nissinen, Sanna Pesonen, Pauliina Toivio and Erja Sormunen in DIGITAL HEALTH
