# Supplementary material for: Exploring the use, usefulness and ease of use of digital occupational health services: A descriptive correlational study of customer experiences
Source: Digit Health. 2024 Apr 9;10:20552076241242668. doi: 10.1177/20552076241242668 (PMC11005500; doi:10.1177/20552076241242668)
Supplement: sj-docx-1-dhj-10.1177_20552076241242668 - Supplemental material for Exploring the use, usefulness and ease of use of digital occupational health services: A descriptive correlational study of customer experiences [file sj-docx-1-dhj-10.1177_20552076241242668.docx]

**Appendix 1. Questionnaire content (Translation from Finnish)**

**BACKGROUND**

1) Gender

1. Male
2. Female
3. Other or I don't want to say.

2) Age

1. Under 20 years
2. 21–30 years
3. 31–40 years
4. 41–50 years
5. 51-60 years
6. 61 years or more

3) Area of residence

1. Uusimaa
2. Kanta-Häme
3. Päijät-Häme
4. Kymenlaakso
5. South Karelia
6. South Savonia
7. Northern Savonia
8. North Karelia
9. Southwest Finland
10. Satakunta
11. Pirkanmaa
12. Central Finland
13. South Ostrobothnia
14. Ostrobothnia
15. Central Ostrobothnia
16. Northern Ostrobothnia
17. Kainuu
18. Lapland

4) Highest level of education

1. Basic education (comprehensive school, secondary school, or elementary school),
2. Upper secondary school or high school graduate
3. Vocational school or course
4. Vocational education at the college level
5. University of applied sciences (polytechnic)
6. University

5) Which of the following best describes your job task?

1. Management (director or supervisor)
2. Occupational safety representative or manager
3. White-collar worker or office employee
4. Blue-collar worker (employee)
5. Someone else, what

6) Skills in information and communication technology are

1. Very good
2. Good
3. Moderate
4. Poor
5. Very poor

7) Workplace’s industry

1. Agriculture, forestry, and fishing
2. Mining and quarrying
3. Manufacturing
4. Electricity, gas, steam, and air conditioning supply
5. Water supply, sewerage, waste management, and remediation activities
6. Construction
7. Wholesale and retail trade; repair of motor vehicles and motorcycles
8. Transportation and storage
9. Accommodation and food service activities
10. Information and communication
11. Financial and insurance activities
12. Real estate activities
13. Professional, scientific, and technical activities
14. Administrative and support service activities
15. Public administration and defense; compulsory social security
16. Education
17. Human health and social work activities
18. Arts, entertainment, and recreation
19. Other service activities

8) Number of employees at the workplace

1. Entrepreneur or 1-4 people
2. 5-9 people
3. 10-49 people
4. 50-249 people
5. 250 people or more

9) Workplace’s occupational health service provider

1. Private medical center
2. Public occupational health service provider (municipality or municipal enterprise)
3. Employer's own occupational health unit
4. Employers' joint occupational health unit
5. Our workplace does not have occupational health services
6. I don't know

10) Do your workplace’s occupational health services include nursing services?

1. Yes
2. No
3. I don’t know

**THE USE OF OCCUPATIONAL HEALTH SERVICES**

11) How often you use occupational health services?

1. Weekly
2. Monthly
3. Less than once a month
4. One a year
5. Less than once a year
6. Not at all

12) How often you use digital occupational health services?

1. Weekly
2. Monthly
3. Less than once a month
4. One a year
5. Less than once a year
6. Not at all

13) Rate how satisfied you are currently with occupational health services (scale 4-10).

14) Rate how satisfied you are currently with digital occupational health services (scale 4-10).

15) Has the COVID-19 pandemic increased your need to use occupational health services?

1. Yes
2. No
3. I don't know

16) Has the COVID-19 pandemic increased your need to use digital occupational health services?

1. Yes
2. No
3. I don't know

17) I used digital occupational health services for the first time

1. Less than a month ago
2. 1-6 months ago
3. 7-12 months ago
4. Over a year ago
5. Never

18) Which of the following digital occupational health services have you used and how often? (scale: 1 = daily, 2= few times a week, 3 = few times a month, 4 = less than once a month, 5= not at all)

*Services for personal customers*

1. Electronic appointment booking
2. Electronic health survey
3. Digital health check-up
4. Digital medical or treatment visit
5. Digital prescription renewal
6. Digital guidance and support
7. Digital work ability negotiation
8. Digital risk test or symptom assessment of health or work ability
9. Digital services for own patient record notes
10. Phone
11. Chat service
12. Email

*Services for employer customers*

1. Digital workplace survey
2. Digital system for monitoring workplace surveys
3. Digital occupational health action plan
4. Digital service for reporting employee information to occupational health care
5. Digital service for reporting staff sick leaves to occupational health care
6. Digital system for monitoring work capacity risks or sick leaves
7. Digital reporting system, for example for tracking costs

19) I have used the digital occupational health services of the following professionals

1. Occupational health physician
2. Occupational health nurse
3. Occupational physiotherapist
4. Occupational health psychologist
5. Expert of social care or work ability coordinator
6. Public health nurse (RN)
7. Nutrition therapist
8. Sleep therapist
9. Memory therapist
10. Someone else, who?

20) Here you can tell which other or whose digital occupational health services you have used. (open-ended question)

21) What new services or digital services would you like your occupational health service to offer you? (open-ended question)

22) Here are statements about digital occupational health services. Mark the box that best represents your experience for each statement. (5-point Likert scales: 1 = completely disagree, 2 = somewhat disagree, 3 = neither disagree nor agree, 4 = somewhat disagree, 5 = completely agree]

1. Many people I know use digital occupational health services.
2. The digital occupational health services are well-known.
3. Digital services are a good idea in occupational health services.
4. Using digital occupational health services has a positive impact on my well-being.
5. I strive to use digital occupational health services whenever possible.
6. I regularly use digital occupational health services.
7. I use multiple digital occupational health services.
8. I recommend the use of digital occupational health services to my colleagues.

23) Here are statements about usefulness of digital occupational health services. Mark the box that best represents your experience for each statement. (5-point Likert scales: 1 = completely disagree, 2 = somewhat disagree, 3 = neither disagree nor agree, 4 = somewhat disagree, 5 = completely agree]

1. Using digital occupational health services improve the quality of the service.
2. Using digital occupational health services give me greater control over my illness.
3. Using digital occupational health services give me greater control over my work ability.
4. Using digital occupational health services help me get the help I need from occupational health professionals even faster.
5. Using digital occupational health services help me get the information I need about health, illness, or work ability even faster.

24) Here are statements about ease of use of digital occupational health services. Mark the box that best represents your experience for each statement. (5-point Likert scales: 1 = completely disagree, 2 = somewhat disagree, 3 = neither disagree nor agree, 4 = somewhat disagree, 5 = completely agree]

1. Learning to use digital occupational health services is easy for me.
2. It is easy to use digital occupational health services.
3. I can use digital occupational health services flexibly according to my needs.
4. The use of digital occupational health services is clear.
5. I can easily find instructions for using digital occupational health services.

25) What else would you like to share regarding digital occupational health services? (open-ended question)

**DEVELOPMENT OF OCCUPATIONAL HEALTH SERVICES**

26) How do you think digital occupational health services should be further developed? (open-ended question)

27) Have you participated in the development of occupational health care services?

1. Yes
2. No
3. No, but I would be interested
